# Ongoing need for education in abortion care

**DOI:** 10.1002/ijgo.14397

**Published:** 2022-08-28

**Authors:** Maeve Eogan, Cliona Murphy, Mary Higgins

**Affiliations:** ^1^ Obstetrics and Gynaecology Rotunda Hospital Dublin Ireland; ^2^ Obstetrics and Gynaecology Royal College of Surgeons of Ireland Dublin Ireland; ^3^ Obstetrics and Gynaecology Coombe Women's and Infants University Hospital Dublin Ireland; ^4^ UCD Perinatal Research Centre, Obstetrics and Gynaecology National Maternity Hospital Dublin Ireland

**Keywords:** abortion, education, legislation

## Abstract

A commentary on the Irish experience of extended abortion care, emphasizing the importance of ongoing medical education.

Four years ago, the Republic of Ireland celebrated a landmark referendum changing the constitution to allow for expanded abortion care. As clinician advocates, we had worked in a system where abortion was restricted, and we spoke out. Extended abortion care started in January 2018, prompting a committed effort by legislators to complete the legislation while in parallel, clinicians and those central to care provision juggled watching the legislation debates with writing guidelines, advising on the models of care, and organizing education.^1^

We realized we already had a wealth of experience in first‐trimester pregnancy loss but needed to transfer these skills to abortion care.^2^ Education was provided to clinical staff from primary to tertiary level care by the Institute of Obstetricians and Gynecologists, the Irish College of General Practitioners, the START doctors group, and the World Health Organization.

More than 3 years later, abortion is now an integrated part of women's health in Ireland. Most abortion care is provided by general practitioners where women receive mifepristone at a face‐to‐face appointment and then take misoprostol home to complete the abortion.^3^ Over 9 weeks of gestation, abortion care takes place in one of the 14 hospitals providing care. After 12 weeks of gestation, abortion is legal if there is a fatal fetal abnormality, a maternal medical condition that would result in a severe risk to the health or life of the mother, or in an emergency. Although there is limited availability of second‐trimester surgical care, we have augmented our holistic second‐ and third‐trimester medical care, including the value of multidisciplinary care, analgesia, support, bereavement, and appropriate spiritual support. Like most countries we adapted with the pandemic to provide telemedicine and remote consultations. We learned to reflect on our own values once actively providing abortion care.^4^

One of the key factors that helped to integrate abortion care was education of students as well as doctors, nurses, midwives, and the wider multidisciplinary team. Education not only includes the technical aspects of abortion care but also the ethical and moral aspects as well as reflections on our own values and experiences. Education of patients is also crucial, including expectation management and safety netting. Currently there is a review of the legislation ongoing. The experience in Ireland has shown that legalization of abortion improves women's health care. The recent Roe reversal in the USA has shown us that as medical professionals we must continue to document and advocate.

## AUTHOR CONTRIBUTIONS

ME, CM, and MH all conceived this commentary topic, and wrote and reviewed the commentary.

## CONFLICT OF INTEREST

The only possible conflict of interest for all three authors would be that all three authors advocated for abortion care within the 2018 referendum. Although we do not believe this to be a conflict of interest, we have had antichoice advocates raise it as an issue in other publications and as such we wished to disclose this.

## DATA AVAILIBILITY STATEMENT

Data sharing is not applicable to this article as no new data were created or analysed in this study.
